# Supplementary material for: Cartilage Collagen Neoepitope C2C Expression in the Articular Cartilage and Its Relation to Joint Tissue Damage in Patients with Knee Osteoarthritis
Source: Biomedicines. 2024 May 11;12(5):1063. doi: 10.3390/biomedicines12051063 (PMC11117959; doi:10.3390/biomedicines12051063)
Supplement: Supplementary file 1 [file biomedicines-12-01063-s001.zip › biomedicines-2887194-supplementary.pdf]

Supplementary Data

Table S1. Patients' characteristics and results of histopathology assessments and C2C staining. F—female patients; M—male patients; LTM—lateral tibial plateau, meniscus-covered area biopsy; MTM—medial tibial plateau, meniscus-covered area biopsy; * Due to technical problems, no MTM sample was obtained from patient 6.

No of patient	Gender	Age (years)	Tibial plateau site	OARSI histo-grade	Macroscopic damage (stage)	OARSI score (grade × stage)	Assessment of C2C staining, %
1	F	60	LTM	6	2	12	57.8
			MTM	3.5		7	93.7
2	M	58	LTM	3.5	3	10.5	28.2
			MTM	1.5		4.5	80
3	F	58	LTM	2.5	3	7.5	87.3
			MTM	2.5		7.5	60.7
4	F	65	LTM	3.5	3	10.5	90.8
			MTM	4.5		13.5	79.2
5	F	61	LTM	2.5	4	10	96.1
			MTM	6		24	97.8
6*	M	59	LTM	2.5	1	2.5	51.7
			MTM	-		-	-
7	F	64	LTM	1.5	3	4.5	74.8
			MTM	4.5		13.5	52.1
8	F	65	LTM	3	3	9	64.7
			MTM	4.5		13.5	99
9	F	65	LTM	4	2	8	16.2
			MTM	3		6	45.6
10	F	55	LTM	2.5	1	2.5	44.4
			MTM	2		2	53.4
11	F	58	LTM	3	2	6	52.9
			MTM	2.5		5	62.8
12	F	59	LTM	1.5	1	1.5	55.6
			MTM	2		2	42.3
13	F	59	LTM	2.5	2	5	88.8
			MTM	4		8	67.5
14	M	58	LTM	2	3	6	47.2
			MTM	5.5		16.5	100
15	F	57	LTM	3	2	6	71.5
			MTM	3		6	79.7
16	F	63	LTM	2	2	4	93.1
			MTM	2.5		5	79.5
17	M	55	LTM	1.5	2	3	50.8
			MTM	4.5		9	74.6
18	M	66	LTM	4.5	3	13.5	86.7
			MTM	5		15	94.4
19	M	65	LTM	3.5	3	10.5	82.5
			MTM	3.5		10.5	91.2
20	M	58	LTM	0	3	0	36.5
			MTM	3		9	100

21	M	62	LTM	4.5	3	13.5	29.5
			MTM	5.5		16.5	80.9
22	F	60	LTM	4	2	8	60.4
			MTM	1.5		3	69.8
23	M	58	LTM	2	2	4	90.2
			MTM	4		8	73.5
24	F	65	LTM	2.5	2	5	44.8
			MTM	4		8	81.2
25	F	56	LTM	3.5	3	10.5	60
			MTM	6		18	99.1
26	F	63	LTM	2	4	8	73.2
			MTM	6.5		26	73.1
27	M	59	LTM	2.5	2	5	69.2
			MTM	3.5		7	95.9
